# Can intraoperative opioid use in hip and knee arthroplasty be reduced further without negatively affecting pain control: A case controlled study

**DOI:** 10.1016/j.jor.2025.01.035

**Published:** 2025-01-31

**Authors:** Adnan Asif, Sam Aktas, Baraniselvan Ramalingam, Hasitha Pananwala, Janna Maier, Femi E. Ayeni, Sol Qurashi S

**Affiliations:** a Dept of Orthopaedics, Nepean Hospital, Derby Street Kingswood, NSW, 2747, Australia; b The Hip and Knee Clinic, 104 Derby St, Penrith, NSW, 2750, Australia; c University of Notre Dame, 160 Oxford Street, Darlinghurst, NSW, 2010, Australia; d Nepean Institute of Academic Surgery, Nepean Clinical School, The University of Sydney. Australia, 62 Derby St, Kingswood, NSW, 2747, Australia

**Keywords:** Opioids, Postoperative nausea and vomiting, Enhanced recovery after surgery, Total hip replacement, Total knee replacement

## Abstract

**Background:**

Whilst forming the backbone of perioperative analgesic regimes in joint replacement surgery, the negative side effect profile of opioids is well known. Common impediments to a smooth running Enhanced Rapid Recovery model of care are often altered cognitive function and postoperative nausea and vomiting (PONV), both related to opioid use.

This study focuses on evaluating whether further reductions in intraoperative opioid use during joint arthroplasty can be safely achieved with minimal impact of such reductions on pain control and postoperative outcomes including opioid requirements and the incidence of PONV and ability to mobilise.

**Method:**

Case controlled review of prospectively collected data assessing intraoperative opioid use, postoperative analgesic requirement, incidence of PONV and cognitive status as well as day 0 mobility postoperatively. 50 patients were randomized in the study and control groups.

**Results:**

The study group received statistically significant lower dose of intraoperative opioids equivalent to 24.18 mg of Morphine compared to control group with equivalent to 69.58 mg of Morphine (p < 0.001). There was no statistically significant increase in analgesia requirement postoperatively and no negative influence on PONV or ability to follow immediate postoperative rehabilitative protocols.

**Conclusion:**

Opioid use intraoperatively can be reduced even further without any compromise of postoperative pain control and PONV and may further reduce impediments to efficiency in rapid recovery models of care.

## 1 Introduction

Effective pain management is a critical component of perioperative care in hip and knee arthroplasty as it directly impacts patient recovery, early mobilization, and overall satisfaction. The administration of intravenous opioids in anaesthesia has traditionally been integral to intraoperative pain management. They also have a role to play in induction of anaesthesia, supplementation of sedation and reduction of immediate postoperative pain and agitation. However, concerns about their adverse effects, such as respiratory depression, increased incidence of postoperative nausea and vomiting (PONV), risk of opioid induced hyperalgesia and postoperative opioid dependency have spurred a shift toward opioid-sparing strategies. PONV and postoperative drowsiness can delay discharge from the hospital, in turn delaying functional recovery and thereby increase medical costs.^1^^,^^2^ These concerns are particularly relevant in the context of the ongoing opioid crisis, where over prescription and misuse of opioids have contributed to a public health emergency characterized by widespread addiction and overdose deaths.^3^ The opioid sparing approach focuses on reducing opioid dosages during surgery without compromising effective pain relief, reflecting a growing interest in balancing efficacy with safety in joint arthroplasty.

Enhanced Recovery After Surgery (ERAS) protocols for joint arthroplasty have further advanced this movement, emphasizing multimodal approaches to perioperative care.^4^ By incorporating non-opioid analgesics, adjuvant therapies, and regional anaesthesia, ERAS protocols seek to optimize pain control while minimizing opioid use. This strategy is particularly valuable in joint arthroplasty, where early mobilization is critical for improving outcomes and minimizing complications such as thromboembolism and joint stiffness.^5^^,^^6^

Multimodal anaesthesia has emerged as a cornerstone of opioid-sparing approaches in joint arthroplasty. This method combines pharmacological agents such as acetaminophen, NSAIDs, COX-2 inhibitors, and adjuvants like ketamine, dexmedetomidine, or gabapentinoids with regional techniques, including peripheral nerve blocks and periarticular injections. These approaches provide targeted, effective analgesia while reducing systemic opioid requirements and the associated risks of PONV, sedation, and delayed recovery.^7^^,^^8^

While reduced opioid use during surgery may decrease immediate side effects such as PONV, it may also result in insufficient intraoperative analgesia, potentially leading to heightened postoperative pain. This could increase the need for postoperative opioid analgesics to manage unrelieved pain, ultimately undermining the goals of opioid-sparing strategies.^9^^,^^10^ Balancing the benefits of reducing intraoperative opioid administration with the risks of inadequate pain control is a critical ingredient in achieving both patients perceived as well as economic benefits of a successful ERAS based model of care. However, the safety and efficacy of further reducing opioid use in joint replacement surgery, and its effects on outcomes such as postoperative opioid consumption, PONV, and early mobilization, require additional research.^9^^,^^11^

Previous studies have shown the role of opioid free anaesthesia in arthroplasty and shown no difference in outcome from opioid sparing approaches.^12^ There are also studies which show that a complete shift to opioid free regimens, albeit well intended, might not be the right solution from the patient's perspective. We need to find a middle group between opioid sparing and opioid free anaesthesia, where the opioids can be minimized to as low as possible where they still exert their effectiveness, while mitigating their side effects.^13^^,^^14^

To our knowledge, none of the previous studies have shown the effect of further reducing intra operative opioids from existing opioid sparing techniques in anaesthesia during arthroplasty surgery. This study focuses on evaluating whether further reductions in intraoperative opioid use during joint arthroplasty can be safely achieved. It also explores the impact of such reductions on postoperative outcomes, including opioid requirements and the incidence of PONV. By addressing these questions, the research aims to contribute to the refinement of ERAS protocols and multimodal analgesic strategies in joint arthroplasty as well as aligning with broader efforts to combat the opioid crisis without compromising enhanced recovery with all its benefits and patient satisfaction. We hypothesized that further reduction in intraoperative opioid use for anaesthesia from the routine opioid sparing anaesthesia techniques will reduce opioid associated side effects without causing a rise in post operative analgesia requirement.

## 2 Materials and methods

This study was carried out by retrospectively analysing prospectively collected data from patients undergoing lower limb arthroplasty surgeries - Total Hip Replacement (THR) and Total Knee Replacement (TKR) between October 2023 and March 2024 at a tertiary level hospital. This study received the approval an institutional review board committee (2024/ETH01787). The inclusion criteria involved patients over 18 years of age, with advanced osteoarthritis of the hip/knee joint, undergoing THR/TKR. Patients with previous history of opioid dependence, medically unfit for surgery, unwilling to participate in the study and those undergoing partial/resurfacing arthroplasty were excluded from the study.

All the surgeries were carried out by the same surgeon and surgical team in the same hospital, following the same surgical technique and perioperative protocols. 100 consecutive arthroplasty procedures were recorded, and patients were randomly allocated to the Study group and the Control group with 50 patients in each group. Our arthroplasty practice focuses on ERAS protocol and maintains opioid sparing anaesthesia as part of routine operative protocols which formed the control group in this study. The patients in the control group were administered the routine opioid sparing anaesthesia by a group of four Anaesthetists during the course of the surgery. In the study group, we aimed to further reduce the administration of intra operative opioid for anaesthesia and was administered by one Anaesthetist. The patients in the control group were administered Fentanyl and its derivatives along with oxycodone as the intra operative opioid. The study group was supplemented with tramadol and a lower dose of Fentanyl. All intraoperative opioids were converted into their equivalent dosage of oral Morphine for standardisation and tabulation. The oral Morphine equivalent of opioids was calculated using the ANZCA opioid calculator.

The surgeries were carried out under general anaesthesia administered through TIVA (Total Intravenous Anaesthesia) and employed the use of multimodal analgesia as part of the protocol. This included preoperative counselling, intraoperative analgesia, local infiltration of anaesthesia in and around the joint and around the surgical incision, postoperative acetaminophen, NSAID's as well as regular and PRN opioids. The postoperative protocol was standardized between both groups and all patients were given the same medications during the course of their hospital stay. This included acetaminophen, NSAID's, regular and PRN synthetic opioid (Topantedol), LMW Heparin, single dose of antibiotic and antiemetic medication. All analgesia was by oral administration after the patient left the operating theatre complex.

Baseline variables collected were age, sex, BMI (Body Mass Index), laterality, joint involvement and length of stay in the hospital. All patients underwent arthroplasty surgery following elective admission via SuperPATH approach for THR and medial parapatellar approach for TKR. In this study, we compared the intraoperative opioid usage between the two groups and to assess if the study group received lower intraoperative opioid and also evaluated the efficacy of opioid sparing anaesthesia and usage of multimodal analgesia. Other outcome measured were the requirement of PRN analgesia post operatively and incidence of nausea and vomiting on Day 0 and Day 1 post operatively and also investigated if any patients had delayed return of cognitive functions and subsequent delayed mobilization post operatively.

### 2.1 Statistical analysis

Continuous variables were expressed as mean, standard deviation, and range and unpaired *t*-test was used to compare these between Study and Control groups. Qualitative nominal data was expressed as percentage and compared between the two groups using the chi-squared test. A p-value of less than 0.05 was considered statistically significant.

## 3 Results

**Fig. 1:**
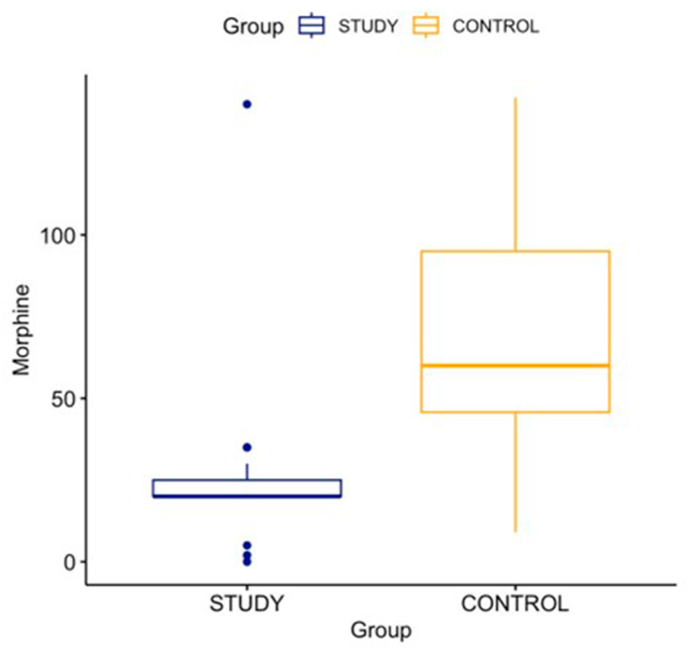
Graphical representation of comparison of mean and range of intra op opioid use between both groups.

Baseline measures of age and sex were similar in both the groups. The BMI in the study group was statistically lesser than the BMI of the control group (29.35 ± 5.72 vs 31.95 ± 7.82, p = 0.04). Laterality and joint involvement were also comparable across both groups. The Length of Stay (LOS) was lower in the study group as compared to the control group (2 ± 0.94 vs 2.46 ± 1.29, p = 0.02) [Table 1]. The primary outcome measure of intraoperative usage of opioids in the study group was equivalent to 24.18 mg of Morphine and was significantly lower than the control group which was equivalent to 69.58 mg of Morphine (p < 0.001). [Table 2] [Fig. 1].

**Table 1:** Comparison of baseline parameters between Study and Control group.

		STUDY (n = 50)	CONTROL (n = 50)	p value
**Age**	Mean (in years)	66.46	66.8	0.4
	SD	10.4	10	
	Range	41–89	25–84	
**Sex**	Male	21 (42 %)	17 (34 %)	0.4
	Female	29 (58 %)	33 (66 %)	
**BMI**	Mean (in kg/m^2^)	29.35	31.95	0.04
	SD	5.7	7.8	
	Range	19.3–42.7	19.6–53.1	
**Laterality**	Right	31 (62 %)	30 (60 %)	0.8
	Left	19 (38 %)	20 (40 %)	
**Joint**	Hip	41 (82 %)	34 (68 %)	0.1
	Knee	9 (18 %)	16 (32 %)	
**LOS**	Mean (in days)	2	2.46	0.02
	SD	0.9	1.3	
	Range	1–4	1–7

**Table 2:** Comparison of mean and standard deviation of intra op opioid use between Study and Control group.

		STUDY GROUP (n = 50)	CONTROL GROUP (n = 50)	p VALUE
**INTRAOP OPIOID**	Mean (mg of Morphine)	24.18	69.58	<0.001
	SD	17.9	36.4

**Fig. 2:**
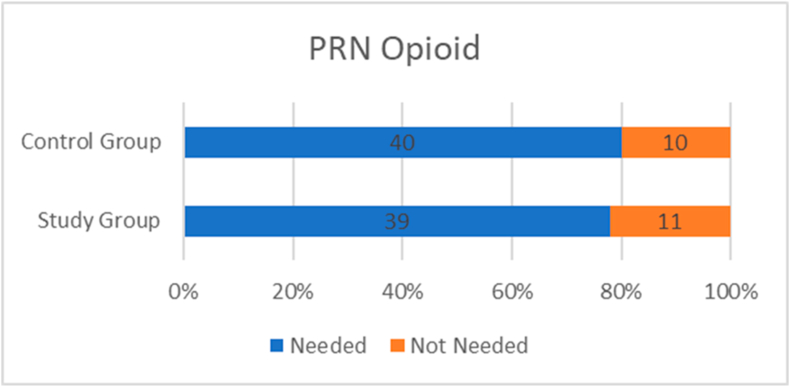
Bar chart showing the requirement of PRN post op opioid use in both groups.

**Fig. 3:**
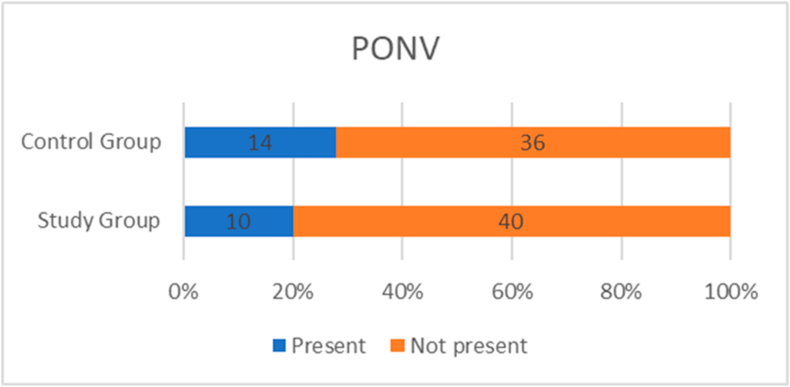
Bar chart showing the frequency of PONV in both groups.

The requirement of PRN opioids for analgesia in the postoperative period was not statistically significant in the study group as compared to the control group (78 % vs 80 %, p = 0.8). The frequency of post operative nausea and vomiting (PONV) on Day 0 and/or Day 1 was not statistically significant in the study group as compared to the control group (20 % vs 28 %, p = 0.1). [Table 3] [Fig. 2][Fig. 3].

**Table 3:** Comparison of frequency of PRN post op opioid use and PONV between Study and Control group.

		STUDY GROUP (n = 50)	CONTROL GROUP (n = 50)	p VALUE	TOTAL
**PRN POST OP OPIOID**	Needed	39 (78 %)	40 (80 %)	0.8	79
	Not needed	11 (22 %)	10 (20 %)		21
**PONV**	Present	10 (20 %)	14 (28 %)	0.1	24
	Not present	40 (80 %)	36 (72 %)		76

All patients in both groups regained their cognitive functions within the first 30 min of reversal of anaesthesia and were mobilized full weight bearing with four-point gait with the help of a walking frame within 4 h post operatively. There were three complications that took place in our study and all the complications were found in the control group. There were two patients who were discharged uneventfully after the surgery and subsequently had dysfunctional cardiac parameters few days post the surgery requiring re-admission.

Both required admission in the ICU and one patient underwent a Coronary Artery Bypass Graft (CABG). Both the patients had previous history of cardiac ailments. One patient had a fall after discharge and sustained a periprosthetic fracture. The patient had undergone a THR and sustained an undisplaced fracture of the greater trochanter. Re-admission was undertaken for observation, analgesia and protected weight bearing. There were no complications in the study group.

## 4 Discussion

This study provides valuable insights into the effects of reducing intraoperative opioid use during joint arthroplasty surgery. The findings underscore the effectiveness of multimodal anaesthesia in achieving adequate pain control while minimizing reliance on opioids. Techniques such as combining local infiltration of anaesthesia (LIA) with non-opioid analgesics have demonstrated promising outcomes in reducing opioid-related side effects and aligning with ERAS protocols. Reducing intraoperative opioids requires careful consideration. Overly aggressive reduction can result in insufficient pain control during surgery, potentially leading to heightened postoperative pain and a greater need for opioids afterward. This issue, likely tied to inadequate analgesia, has been observed in previous studies and warrants further investigation.^9^

This study found a middle ground between opioid sparing anaesthesia and opioid free anaesthesia. We assessed whether further reducing intraoperative anaesthetic opioids can maintain post operative analgesia, while reducing the opioid associated complications. The primary outcome measurement showed that the study group did get a statistically significant reduction in their intraoperative opioids as compared to the patients in the control group. This finding supported our hypothesis that aimed to reduce the amount of opioids given in the study group as well as finding a balance between reduced opioids intra operatively, without being completely devoid of opioids.

The baseline characteristics of age, sex, laterality and joint involvement were similar in both groups. The length of stay was statistically lower in the study group, and this shows that patients in the study group had quicker immediate post operative recovery and hence were eligible for earlier discharge. The BMI in the study group was statistically lower than in the control group. The effect of this variable is unclear but not likely to bias the result. If anything, it would support our findings that intraoperative opioid reduction can be achieved without compromising postoperative pain control. This is due to the fact that opioid metabolism would be quicker in patients with a lower BMI.

A study Laura et al.^15^ showed that intra operative opioid usage has a distinct correlation to the post operative analgesia requirement and reducing intra operative opioids can increase post operative pain and analgesia requirement. In our study, despite receiving lower opioids, the patients in the study group had similar postoperative analgesia requirements as the control group. The patients in the study group did not require any additional post operative opioids for analgesia. Elvir-Lazo et al.^16^ in their study showed that intraoperative opioids have a significant role to play in PONV and the use of multimodal anaesthesia techniques can reduce the incidence and lead to enhanced recovery. In our study there was no statistically significant difference in the incidence of PONV between the two groups. All patients in the study were cognisant within the first hour after surgery and were able to be mobilized as planned. Hence, reducing intraoperative opioids did not affect the immediate rehabilitation (due to pain control issues) and mobilization and in turn conformed with the protocols of enhanced recovery.

Postoperative recovery and pain management are intricately linked to intraoperative practices, making this a critical area of focus in joint arthroplasty. While reducing intraoperative opioids can help decrease the incidence of postoperative nausea and vomiting (PONV), our results suggest that careful patient monitoring and personalized approaches to pain management are essential. Factors such as individual pain thresholds and pre-existing opioid tolerance may influence postoperative outcomes. PONV can delay recovery and impede early mobilization, which are key objectives in hip and knee arthroplasty and these findings reinforce the potential advantages of limiting opioid use while maintaining effective pain control.^7^^,^^10^ The reduction in PONV observed among patients who received lower doses of intraoperative opioids further supports the benefits of an opioid-sparing approach.

The concepts like ‘opioid induced hyperalgesia’ (OIH), a paradoxical state whereby increased nociceptive sensitization occurs as a consequence exposure to opioids has been well described.^20^ Avoiding OIH would be another incentive in aiming for an overall opioid reduction in the short term but also longer term, in context of the ongoing opioid crisis, our findings carry significant implications beyond the surgical setting. The misuse and over prescription of opioids have contributed to a public health challenge marked by rising rates of dependency and overdose. By adopting multimodal pain management strategies, healthcare providers can address the dual goals of improving immediate patient outcomes and reducing the risk of long-term opioid use. There were no surgical or anaesthetic related complications in our study. The three complications that took place all occurred after the patient had been discharged and the events took place outside the hospital, requiring re-hospitalisation.

A meta-analysis by Zhang et al.^17^ for opioid free anaesthesia in gynaecological surgeries, showed that reducing intraoperative opioids can reduce the incidence of PONV, but it did not affect the incidence of post operative pain. Our study shows similar results in arthroplasty surgeries. Meta-analysis by Olausson et al.^18^ and Frauenknecht et al.^19^ conducted which compared opioid-free to opioid sparing anaesthesia and they showed that while opioid-free anaesthesia reduced PONV, it did not have a significant difference in the post operative pain profile as compared to opioid sparing techniques. These studies were not focused on any subspecialty surgery and encompassed all types of surgical procedures. Chassery et al.^12^ in their RCT showed that opioid-free anaesthesia does not provide any significant improvement from opioid sparing anaesthesia and a complete avoidance of opioids intraoperatively was uncalled for. To the best of our knowledge, our study is the first which assesses the impact of reduced intraoperative opioids in arthroplasty surgery.

There were some limitations to our study. The study was carried out in a single centre. We had a small sample size of 50 patients in each group and this can lead to a type II error in statistical analysis. We had a single anaesthetist in the study group whereas there were four different anaesthetists in the control group, and this may induce a bias. However, the treatment and evaluation protocols were uniform and standardized in both the groups, which would mitigate the bias. Another limitation of this study was that we only assessed two postoperative outcomes and for a short perioperative period. Further multicentre studies with larger sample size need to be carried out to substantiate the results and evidence.

## 5 Conclusion

This study has opened a new vista for the intraoperative opioid use in patients undergoing joint replacement surgery. Future research should examine the long-term effects of reduced intraoperative opioid use, focusing on chronic pain management, as well as functional recovery and patient satisfaction. Furthermore, exploring innovative approaches such as long-acting regional anaesthesia and emerging non-opioid analgesics may offer new opportunities to optimize pain control and recovery. By refining pain management protocols in joint arthroplasty, we can better align with both clinical and societal imperatives, ensuring safer and more effective care for patients.^3^

## CRediT authorship contribution statement

**Adnan Asif:** Conceptualization, Methodology, Formal analysis, Writing – review & editing. **Sam Aktas:** Writing – review & editing, All authors read and approved the final manuscript. **Baraniselvan Ramalingam:** Formal analysis. **Hasitha Pananwala:** Data curation. **Janna Maier:** Data curation. **Femi E. Ayeni:** Methodology, Formal analysis. **Sol Qurashi S:** Conceptualization, Methodology, Formal analysis, Data curation, Writing – review & editing.

## Statement of ethics

This study received the approval of Nepean Blue Mountains Local Health District (NBMLHD) Human Research Ethics Committee (HREC) 2024/ETH01787 and performed in accordance with the ethical standards laid down in the 1964 Declaration of Helsinki.

## Funding statement

This research did not receive any specific grant from funding agencies in the public, commercial or not-for-profit sectors.
